# Dataset on structure-antioxidant activity relationship of active oxygen catalytic lignin and lignin-carbohydrate complex

**DOI:** 10.1016/j.dib.2019.104413

**Published:** 2019-08-17

**Authors:** Bo Jiang, Yu Zhang, Huifang Zhao, Tianyu Guo, Wenjuan Wu, Yongcan Jin

**Affiliations:** a Jiangsu Co-Innovation Center of Efficient Processing and Utilization of Forest Resources, Nanjing Forestry University, Nanjing, 210037, China; b Institute of Botany, Jiangsu Province and the Chinese Academy of Sciences, Nanjing, 210014, China

**Keywords:** Rice straw, Lignin, Lignin-carbohydrate complex, Structure, Antioxidant activity

## Abstract

The data presented in this article are related to the research article entitled “Structure-antioxidant activity relationship of active oxygen catalytic lignin and lignin-carbohydrate complex” (Jiang et al.). It supplements the article with thermostability of milled wood lignin (MWL) and alkali-oxygen lignin (AOL), main substructures of lignin in rice straw, main products and yield of nitrobenzene oxidation of lignin-carbohydrate complexes (LCCs), Fourier transform infrared spectroscopy of LCCs, radical (ABTS·) scavenging ability of lignins and signal assignment of lignins and LCCs in nuclear magnetic resonance spectra (^1^H, ^13^C, 2D HSQC NMR). The dataset is made publicly available and can be useful for extending the structural and bioactive research and critical analyses of lignin and LCC.

**Table:** Specifications Table

Subject	Agricultural and Biological Sciences (General)
Specific subject area	Structure-antioxidant activity relationship of lignin
Type of data	Tables Figures
How data were acquired	Thermostability (thermogravimetric analyzer, SDT 650, USA), nitrobenzene oxidation (gel chromatography, Shimadzu Co., Kyoto, Japan) equipped with a flame ionization detector and SH-Rtx-5 column (Shimazu Co., Kyoto, Japan), Fourier transform infrared spectroscopy (VERTEX 80 V FTIR spectrometer, Bruker, Germany), radical scavenging ability (microplate spectrophotometer, Infinite M200, Kunshan, China), nuclear magnetic resonance spectra (NMR; AVANCE III 600 MHz instrument, Bruker, Switzerland).
Data format	Raw data, Analyzed data
Parameters for data collection	Parameters of alkali-oxygen treatment were formulated and fine-tuned according to the manufacturing technique of the pulp mill in Jiangsu. Parameters of nitrobenzene oxidation and NMR refer to the published papers [2], [3].
Description of data collection	The data in this article were recorded and collected from the software of corresponding detecting instruments.
Data source location	Nanjing, Jiangsu, China
Data accessibility	Data is available with this article
Related research article	B. Jiang, Y. Zhang, H. Zhao, T. Guo, W. Wu, Y. Jin, Structure-Antioxidant Activity Relationship of Active Oxygen Catalytic Lignin and Lignin-Carbohydrate Complex. International Journal of Biological Macromolecules

**Table:** 

**Value of the data** • Data are convenient to examine the structural characteristics of milled wood lignin and alkali-oxygen lignin from rice straw and are useful to compare similar studies using other lignocelluloses as feedstocks. • The data throw light on the structure-antioxidant relationship and the molecular mechanism of lignin, which will greatly move forward the value-added applications of lignin. • Data can guide the usage of lignin from pulp mills on agriculture and polymeric materials.

## 1 Data

**Fig. 1:**
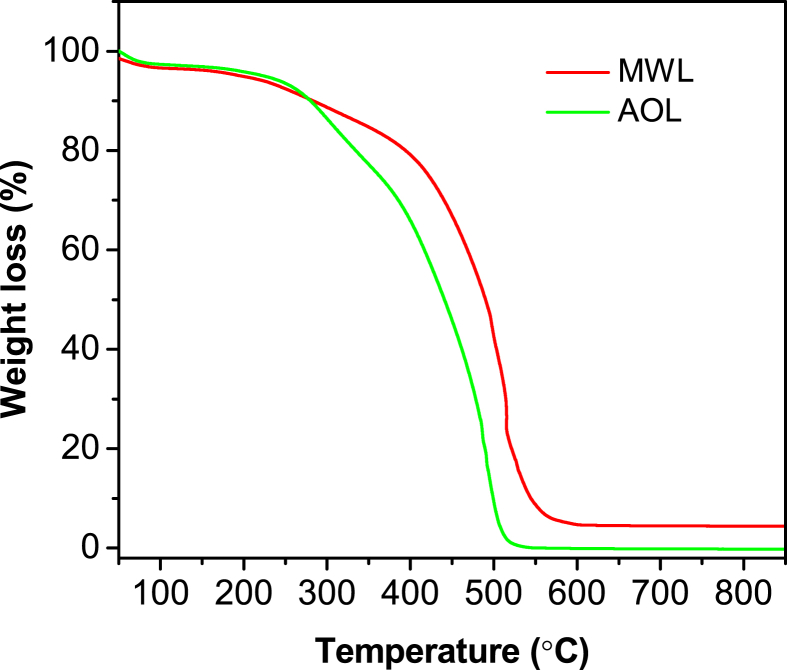
The weight loss of MWL and AOL with temperature.

**Fig. 2:**
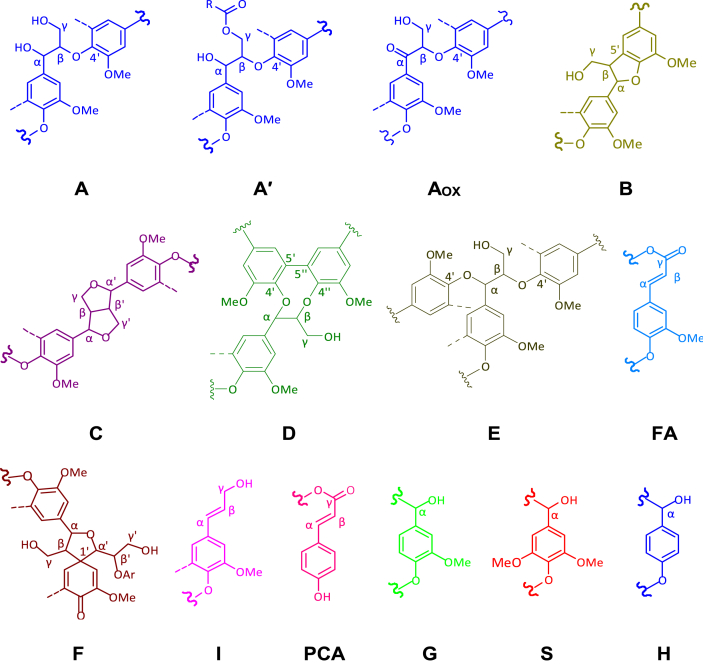
Main substructures of lignin in rice straw: (A) *β*-O-4′ linkages with a free –OH at C_*γ*_; (A′) *β*-O-4′ linkages with acetylated and/or *p*-hydroxybenzoated –OH at C_*γ*_; (Aox) *β*-O-4′ linkages with a free –OH at C_*γ*_ and a C_*α*_ = O; (B) phenylcoumaran substructures formed by *β*-5′ and *α*-O-4′ linkages; (C) resinol substructures formed by *β*-*β*′, *α*-O-*γ*′ and *γ*-O-*α*′ linkages; (D) dibenzodioxocin substructures formed by *β*-O-4′ and *α*-O-4′ linkages; (E) *α*-O-4′ and *β*-O-4′ linkages with a free –OH at C_*γ*_; (F) spirodienone substructures formed by *β*-1′ and *α*-O-*α*′ linkages; (FA) ferulate substructures; (I) cinnamyl alcohol end-groups; (PCA) *p*-coumarate substructures; (G) guaiacyl units; (S) Syringyl units; (H) *p*-hydroxyphenyl units.

**Fig. 3:**
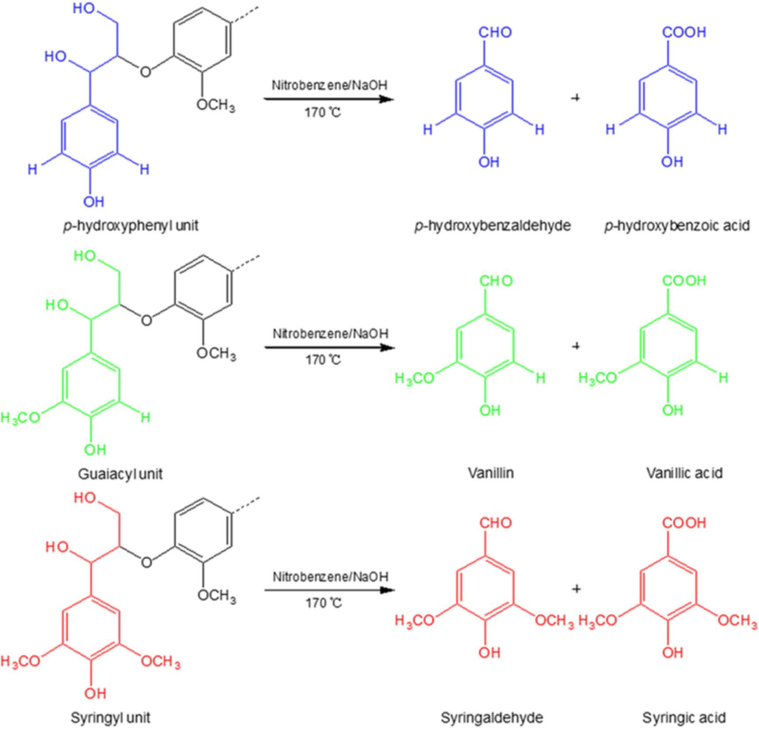
The main products of alkali nitrobenzene oxidation of lignin.

**Fig. 4:**
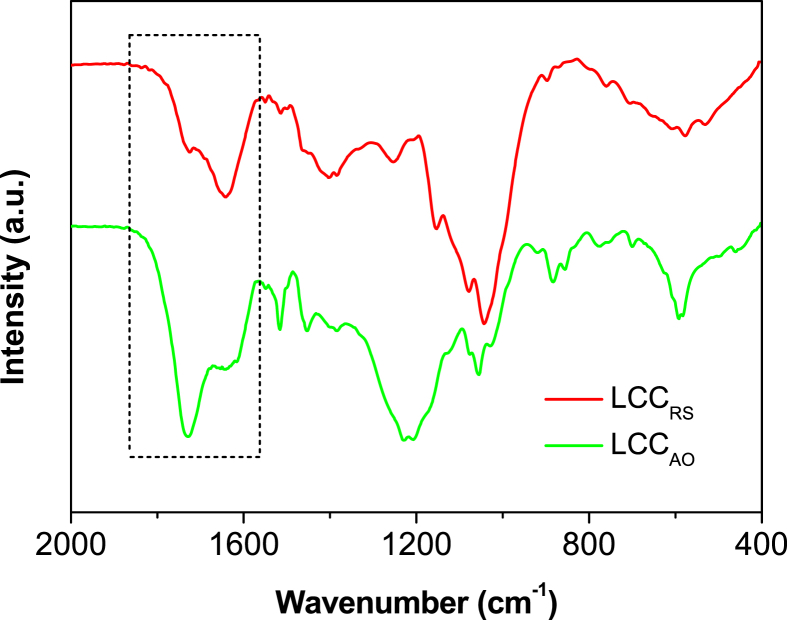
FTIR spectra of LCCs.

**Fig. 5:**
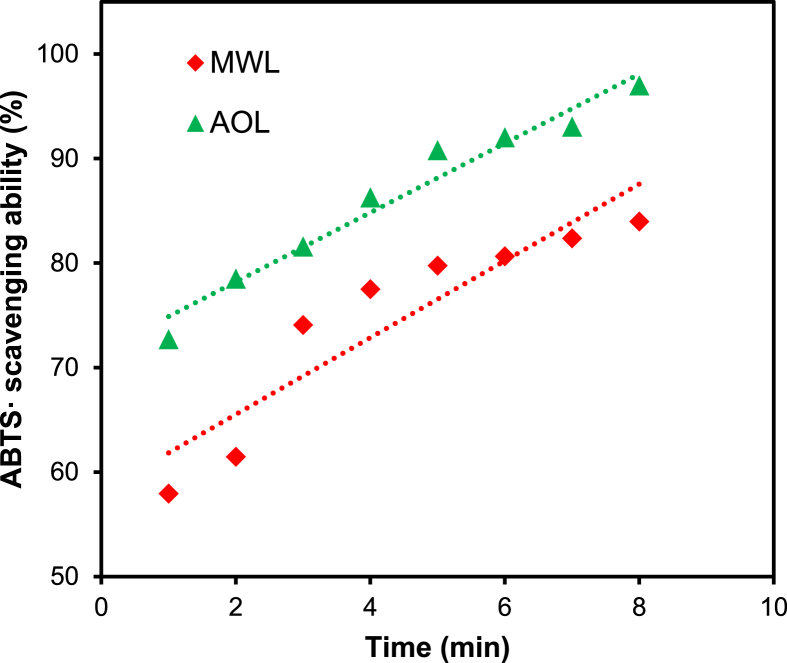
The ABTS· scavenging ability of MWL and AOL.

In this report, we present data on the structure-antioxidant activity relationship of lignin and LCC to supplement the analysis of our research article [1]. Thermostability is an important property of antioxidants to identify its antioxidant capacity, which was demonstrated by TGA as shown in Fig. 1. Spectroscopic methods (NMR and FTIR) combined with chemical degradation (nitrobenzene oxidation) can give comprehensive structural analysis of lignin and LCC. The signal assignment of NMR (Table 1, Table 2, Table 3) and FTIR (Table 5 and Fig. 4) spectra supplements the information of the main substructures (Fig. 2) of lignin in rice straw, which can be assigned and analyzed according to the published literatures [4], [5], [6], [7]. Chemical degradation of nitrobenzene oxidation (Fig. 3) endows this research with monomeric composition and the condensation degree of lignin, and the raw data were listed in Table 4. The assessment of ABTS· scavenging ability (Fig. 5) is used to prove the data of corresponding DPPH· assay and to demonstrate that the AOL has higher antioxidant activity.

**Table 1:** Signal assignment for ^1^H NMR spectra of MWL and AOL.

Label	*δ*_H_ (ppm)	Assignment
1	7.42–8.00	Aromatic proton in *p*-hydroxyphenyl units
2	6.75–7.42	Aromatic proton in guaiacyl units
3	6.15–6.75	Aromatic proton in syringyl units
4	5.69–6.15	H_*α*_ in *β*-O-4′ and *β*-1′ structure
5	5.22–5.69	H_*α*_ in *β*-5′ and *α*-O-4′ structure
6	4.48–5.22	H_*α*_ in *β*-*β*′ structure
7	4.01–4.48	H_*γ*_ in *β*-O-4′ structure
8	3.43–4.01	Proton in methoxyl
9	2.15–2.42	Proton in aromatic acetates
10	1.58–2.15	Proton in aliphatic acetates
11	0.66–1.58	Proton in –CH_2_– and –CH_3_

**Table 2:** Signal assignment for^13^C NMR spectra of MWL and AOL.

*δ*_C_ (ppm)	Assignment	*δ*_C_ (ppm)	Assignment
166.5	C_9_ in p-coumarates	128.0	C_*α*_ and C_*β*_ in Ar-CH=CH–CH_2_OH
160.0	C_4_ in p-coumarates	125.9	C_5_/C_5’_ in non-etherified 5-5′ units
156.4	C_4_ in p-hydroxyphenyl units	125.1	C_1_ in *p*-coumarates
152.9	C_3_/C_3’_ in etherified 5-5 units, C_*α*_ in –CH=CH–CHO units	123.0	C_6_ in ferulates
152.5	C_3_/C_5_ in etherified syringyl units and guaiacyl ring of 4-O-5′ units	122.6	C_1_ and C_6_ in Ar–C (=O)C–C unis
151.3	C_4_ in etherified guaiacyl units with *α*-C=O	119.4/118.4	C_6_ in guaiacyl units
149.7	C_3_ in etherified guaiacyl units	115.1/114.7	C_5_ in guaiacyl units
148.4	C_3_ in guaiacyl units	111.1/110.4	C_2_ in guaiacyl units
146.8	C_4_ in etherified guaiacyl units	106.8	C_2_/C_6_ in syringyl units with α-C=O
145.8	C_4_ in non-etherified guaiacyl units	104.3	C_2_/C_6_ in S syringyl units
145.0	C_4_ in etherified 5-5′ units	86.6	C_*α*_ in guaiacyl type *β*-5′ units
143.3	C_4_ in non-etherified 5-5′ units	84.6	C_*β*_ in guaiacyl type *β*-O-4′ units (threo)
138.2	C_4_ in syringyl etherified units	83.8	C_*β*_ in guaiacyl type *β*-O-4′ units (erythro)
134.6	C_1_ in etherified syringyl and guaiacyl units	72.4	C_γ_ in *β*-*β*′ and *β*-aryl ether
133.4	C_1_ in non-etherified syringyl and guaiacyl units	71.2	C_*α*_ in guaiacyl type *β*-O-4′ units (threo)
132.4	C_5_ in etherified 5-5′ units	63.2	C_*γ*_ in guaiacyl type *β*-O-4′ units with *α*-C=O
131.1	C_1_ in non-etherified 5-5′ units	62.8	C_*γ*_ in guaiacyl type *β*-5′, *β*-1′ units
130.3	C_2_/C_6_ in p-coumarates	60.2	C_*γ*_ in guaiacyl type *β*-O-4′ units
129.3	C_*β*_ in Ar-CH=CH–CHO	55.6	C in Ar-OCH_3_
128.1	C_2_/C_6_ in p-hydroxyphenyl units	29.2	CH_2_ in aliphatic side chain

**Table 3:** Assignment of the polysaccharide signals in the 2D HSQC NMR spectra of LCCs.

Label	*δ*_C_/*δ*_H_ (ppm)	Assignment
Est	66–62/4.5–4.0	C–H in *γ*-ester linkages
X_5_	62.9/3.41	C_5_–H_5_ in *β*-D-xylopyranoside
X_2_	72.7/3.05	C_2_–H_2_ in *β*-D-xylopyranoside
X2_2_	73.1/4.50	C_2_–H_2_ in 2-O-acetyl-*β*-D-xylopyranoside
X_3_	73.7/3.29	C_3_–H_3_ in *β*-D-xylopyranoside
X3_3_	74.9/4.81	C_3_–H_3_ in 3-O-acetyl-*β*-D-xylopyranoside
X_4_	75.5/3.53	C_4_–H_4_ in *β*-D-xylopyranoside
BE_1_	81.6/4.63	C_*α*_-H_*α*_ in benzyl ether (secondary OH of carbohydrate) linkages
Ara_4_	86.8/4.32	C_4_–H_4_ in arabinan
*α*X_1(R)_	92.5/4.89	C_1_–H_1_ in (1→4)-*α*-d-xylopyranoside (R)
*β*X_1(R)_	97.6/4.25	C_1_–H_1_ in (1→4)-*β*-d-xylopyranoside (R)
X23_1_	99.5/4.74	C_1_–H_1_ in 2,3-O-acetyl-*β*-d-xylopyranoside
X2_1_	99.8/4.52	C_1_–H_1_ in 2-O-acetyl-*β*-d-xylopyranoside
X3_1_	101.9/4.28	C_1_–H_1_ in 3-O-acetyl-*β*-d-xylopyranoside
PhGlc_1_	100.3/5.09	C_1_–H_1_ in phenyl glycoside linkages
PhGlc_3_	101.9/4.95	C_3_–H_3_ in phenyl glycoside linkages
X_1_/Glc_1_	103.2/4.29	C_1_–H_1_ in *β*-d-xylopyranoside/*β*-d-glucopyranoside

**Table 4:** The yield and ratio of nitrobenzene oxidation products of LCCs.

Samples	Yield (mmol/g-lignin)				V/S/H^a^
	V	S	H	Total	
LCC_RS_	1.20 ± 0.01	0.41 ± 0.01	0.46 ± 0.00	2.07 ± 0.02	58/20/22
LCC_AO_	0.22 ± 0.00	0.18 ± 0.03	0.18 ± 0.01	0.58 ± 0.01	38/31/31

a V = vanillin + vanillic acid; S = syringaldehyde + syringic acid; H = *p*-hydroxybenzaldehyde + *p*-hydroxybenzoic acid.

**Table 5:** The position and assignment of absorption peaks in LCCs.

Wavenumber (cm^−1^)	Assignment
1724	Stretching vibration of non-conjugate C=O
1641	Stretching vibration of conjugate C=O
1505	Stretching vibration of benzene ring
1462	Bending vibration of C–H (CH_2_, CH_3_)
1401	Stretching vibration of benzene ring
1263	Stretching vibration of C–O in G-unit
1160	Stretching vibration of phenolic acid ester
1086	Bending vibration of C–H and C–O
840	Out-of plane bending vibration of C–H in benzene ring (S/H)

## 2 Experimental design, materials, and methods

### 2.1 Thermostability and FTIR

The thermostability was determined by a thermogravimetric analyzer (SDT 650) using a heating rate of 5 °C/min in air from room temperature to 1000 °C.

FTIR spectra of LCCs were recorded using a FTIR spectrometer (VERTEX 80 V, Bruker, Germany). 1 mg of samples was mixed with 200 mg of KBr. After grinding and tabletting, the FTIR spectra was recorded with the scan resolution of 4 cm^−1^ and the scan area of 4000−400 cm^−1^.

### 2.2 NMR characterization

MWL and AOL were acetylated according to the method reported by Lu and Ralph [8] for the determination of ^1^H and ^13^C NMR. 20 mg of acetylated lignins was dissolved in 0.5 mL DMSO-*d*_6_ for ^1^H NMR detection. For the quantitative ^13^C NMR experiment, acetylated lignin (150 mg) was dissolved in DMSO-*d*_6_ (0.5 mL). Chromium (III) acetylacetonate (20 μL, 0.01 M) was added to provide complete relaxation of all nuclei. The mixture was then transferred to a Shigemi microtube and characterized at 25 °C. The acquisition parameters were: 90° pulse width, a relaxation delay of 1.7 s, and an acquisition time of 1.2 s. A total of 20,000 scans were collected.

For 2D HSQC NMR test of LCCs, the LCC samples (50 mg) were dissolved in 0.5 mL of DMSO-*d*_6_. The number of collected complex points was 2048 for the ^1^H-dimension with a recycle delay of 1.5 s. The number of transients was 64, and 256 time increments were recorded in the ^13^C-dimension. The ^1^J_CH_ used was 145 Hz. Processing used typical matched Gaussian apodization in the ^1^H-dimension and squared cosine-bell apodization in the ^13^C-dimension. Prior to Fourier transformation, the data matrices were zero-filled to 1024 points in the ^13^C-dimension.

### 2.3 Nitrobenzene oxidation

Nitrobenzene oxidation was applied to the LCCs according to the procedure reported by Chen [2]. Briefly, 10 mg of sample was reacted with 0.25 mL nitrobenzene in a stainless steel bomb at 170 °C for 2 h under alkali condition (4 mL 2 mol/L sodium hydroxide). Then, the bomb was cooled in cold water immediately and 1 mL 0.1 mol/L sodium hydroxide solution containing 3-ethoxy-4-hydroxybenzaldehyde (0.3 g/L) was added as the internal standard. The mixture was extracted three times with dichloromethane in separating funnel. The aqueous phase was acidified with 4 mol/L HCl to pH = 1 and extracted twice with dichloromethane and once with ethyl ether. The combined organic phase was extracted with 20 mL deionized water and the organic phase was mixed with anhydrous sodium sulfate overnight. After removing the insoluble inorganic materials by filtration, the solution was evaporated to dryness and silylated using N,O-bis(trimethylsilyl) acetamide at 100 °C for 10 min. The silylated samples were analyzed by gas chromatography (Plus 2010) equipped with a flame ionization detector and SH-Rtx-5 column (Shimazu Co., Kyoto, Japan).

### 2.4 Assessment of DPPH·and ABTS·scavenging ability

The DPPH· and ABTS· radical scavenging assay of lignins and LCCs was performed using a spectrophotometric method. Samples were dissolved in 90% 1,4-dioxane/water (v/v). The DPPH· was dissolved in anhydrous ethanol with the concentration of 6 × 10^−5^ mol/L. ABTS· was generated by reacting 2,2′-Azino-bis (3-ethylbenzothiazoline-6-sulfonic acid) diammonium salt (7 mM) with 2.45 mM potassium persulfate (K_2_S_2_O_8_) in ultrapure water and then letting the solution stand for 15 h in the dark at room temperature. The radical solution was adjusted to obtain an UV absorbance of 0.70 ± 0.02 at 517 nm and 734 nm for DPPH· and ABTS·, respectively. The concentration of lignin and LCCs in tested sample is 0.03 mg/mL. The absorbance of tested sample was measured using a microplate spectrophotometer (Infinite M200, Ku nshan, China). The radical scavenging ability was calculated using the following formula:

Scavenging ability (%) = [1-(A_i_-A_j_)/A_0_]*100

where A_i_ is the absorbance of the tested sample; A_j_ is the absorbance of the blank sample via anhydrous ethanol replacing DPPH· or ultrapure water replacing ABTS· solution; A_0_ is the absorbance of the blank sample via anhydrous ethanol or ultrapure water replacing lignin solution.
